# Eating Disorders and Major Depression: Role of Anger and Personality

**DOI:** 10.1155/2011/194732

**Published:** 2011-10-02

**Authors:** Abbate-Daga Giovanni, Gramaglia Carla, Marzola Enrica, Amianto Federico, Zuccolin Maria, Fassino Secondo

**Affiliations:** Eating Disorders Program, Section of Psychiatry, Department of Neuroscience, University of Turin, Via Cherasco 11, 10126 Turin, Italy

## Abstract

This study aimed to evaluate comorbidity for MD in a large ED sample and both personality and anger as clinical characteristics of patients with ED and MD. We assessed 838 ED patients with psychiatric evaluations and psychometric questionnaires: Temperament and Character Inventory, Eating Disorder Inventory-2, Beck Depression Inventory, and State-Trait Anger Expression Inventory. 19.5% of ED patients were found to suffer from comorbid MD and 48.7% reported clinically significant depressive symptomatology: patients with Anorexia Binge-Purging and Bulimia Nervosa were more likely to be diagnosed with MD. Irritable mood was found in the 73% of patients with MD. High Harm Avoidance (HA) and low Self-Directedness (SD) predicted MD independently of severity of the ED symptomatology, several clinical variables, and ED diagnosis. Assessing both personality and depressive symptoms could be useful to provide effective treatments. Longitudinal studies are needed to investigate the pathogenetic role of HA and SD for ED and MD.

## 1. Introduction

Lifetime comorbidity between Eating Disorders (EDs) and Mood Disorders has been confirmed by several retrospective studies reporting that in Anorexia Nervosa (AN) the prevalence of mood disorders varies between 64.1% and 96% whereas in Bulimia Nervosa (BN) between 50% and 90%. In addition, a substantial part of individuals affected by an ED is likely to be affected also by a mood disorder, and the current comorbidity varies from 12.7 to 68% in AN and is about 40% in BN [1]. Major Depression (MD) is the most prevalent comorbid mood disorder in ED patients, and the severity of depressive symptomatology seems to be related to the ED one [2–5].

In spite of the importance of this topic, most of previous studies on mood in ED were conducted on small samples (e.g., fewer than 30 cases), and the role of age, duration of illness, and weight were not considered. Moreover, ED subtypes and their differences were not carefully classified, particularly Eating Disorder Not Otherwise Specified (EDNOS) [6], and the dimensional assessment of depressive symptomatology wasnot evaluated in detail. Furthermore, the experience and expression of anger in patients with comorbid depression and ED have been relatively neglected, even though hostility and aggressiveness are commonly reported in ED populations [7, 8]. 

Indeed anxious/preoccupied behaviors, mood intolerance, and dysthymic traits have been reported in ED patients [5, 9, 10]. Studies conducted with the Temperament and Character Inventory (TCI) [11] have found that ED individuals both in the acute phase [9, 12, 13] and after remission [14–16] performed higher scores of Harm Avoidance (HA) and low scores of Self-Directedness (SD) than healthy controls. Individuals with these personality features are thought to have poorer abilities to cope with stressful life events [9, 13] and, although future studies are needed high HA and low SD have been proposed as potential risk factors for ED and not only consequences of the illness [13]. Various authors have found such alterations of personality dimensions—high HA and low SD—in patients with MD [17–19] also after remission [20–22]: hence, it should be noted that the alterations of these traits are not only state dependent, as suggested by some studies [23]. Despite these findings, few studies have examined the personality traits of patients with comorbid ED and MD, after controlling for eating psychopathology and other clinical variables. 

With this study we aimed to (a) evaluate the prevalence of a current MD in a large sample of ED patients; (b) assess the prevalence of MD with irritable mood in ED patients; (c) provide data supporting the correlation between MD and ED severity; (d) show possible differences between ED patients with and without MD, independently from the severity of eating symptomatology.

## 2. Materials and Methods

The sample consisted of 838 patients admitted to the outpatient service of the ED Program of the University of Turin between the 1st of January 2003 and the 31st of December 2010. All subjects were diagnosed with an ED, and the sample was represented by the following subjects: AN, restricting type (AN-R), *n* = 214; AN, binge-purging type (AN-BP), *n* = 103; BN, purging type (BN-E), *n* = 223, Eating Disorder Not Otherwise Specified (EDNOS), *n* = 298. Patients with BN, nonpurging type, were excluded because their number (*n* = 13) was not statistically relevant. Diagnoses of ED and MD were based on the structured clinical interview for DSM-IV (SCID-I) [24]. Exclusion criteria were medical comorbidity (e.g., epilepsy or diabetes), drug abuse, and male gender. 

The first two assessment interviews were conducted by psychiatrists experienced in the diagnosis and treatment of ED. Irritable mood and angry outbursts were assessed according to the criteria proposed by Fava and Kellner [25] and evaluated with clinical interviews derived by authors' questionnaires. Patients completed the self-report questionnaires described below between the first and the second interview. After complete description of the study to the subjects, written informed consent was obtained. The Italian version of self-rating instruments was used.

### 2.1. Temperament and Character Inventory (TCI)

The TCI [11] is divided into seven dimensions. Four of these assess temperament (Novelty Seeking [NS], Harm Avoidance [HA], Reward Dependence [RD], and Persistence [P]), defined as partly heritable emotional responses, stable throughout life, mediated by neurotransmitters in the central nervous system. The other three dimensions assess character (Self-Directedness [SD], Cooperativeness [C], and Self-Transcendence [ST]), defined as the overall personality traits acquired through experience.

### 2.2. State-Trait Anger Expression Inventory (STAXI)

The 44-item STAXI [26] measures the intensity of anger as an emotional state (State-anger) and the disposition toward anger as a personality trait (Trait-anger). Anger Expression-In (AX-In) measures the suppression of angry feelings. Anger Expression-Out (AX-Out) measures the frequency of the expression of anger toward other people or objects in the environment. Anger Expression Control (AX-Con) measures the control of anger. AX/Ex provides a general index of the expression of anger.

### 2.3. Beck Depression Inventory (BDI)

The BDI [27] is a self-report questionnaire used to assess the severity of symptoms of depression. Clinical euthymia is defined by scores lower than 10. The BDI has been found to be a reliable instrument for assessing depressive symptoms in ED patients.

### 2.4. Eating Disorder Inventory-2 (EDI-2)

The EDI-2 [28] is a self-report measure of disordered eating attitudes and behaviors, as well as of personality traits common to individuals with ED. Eleven subscales evaluate symptoms and psychological correlates of ED.

### 2.5. Statistical Analysis

Statistical analyses were carried out using Statistical Package for Social Sciences (SPSS) software version 13.0 for Windows (SPSS 13.0 Application Guide. Chicago, SPSS, Inc., 2004). Categorical data were compared using the chi-squared test, and continuous data were analysed using a two-tailed independent *t*-test. Age, age of onset of the disorder, duration of illness, and Body Mass Index (BMI) were analysed in terms of confounding variables using a Univariate General Linear Model.

A logistic regression analysis was performed to detect personality variables that independently relate with MD. The presence/absence of MD was regarded as a dependent variable. ED diagnosis, duration of illness expressed in months, BMI, age, age of onset of the disorder, presence/absence of irritable mood, and scores on the TCI, EDI-2, and STAXI scales were included as independent variables. 

To assess the possible correlation with the depressive state of personality traits we found as significant at the linear regression has been checked the linear correlation (Pearson bivariate) between BDI and personality score and we performed also a MANOVA with personality scores as dependent variables, depressive versus nondepressive group as fixed factor, the BDI score as covariate, an the BDI group interaction.

## 3. Results

### 3.1. Sociodemographic and Clinical Features of the Sample

Sociodemographic and clinical features are reported in Tables 1 and 2.

### 3.2. MD Diagnosis and Depressive Symptomatology

Subjects with MD represent the 19.5% (*n* = 161) of the sample: 15.3% of AN-R (*n* = 33), 25.5% of AN-BP (*n* = 25), 25.3% of BN (*N* = 56), and 16% of EDNOS (*n* = 47). Significant differences were found among AN and EDNOS individuals and the other ED subtypes (*χ* ^2^ = 11.752; *P* = 0.008).

Patients with MD did not show any significant difference when compared to those without MD in regard to age, age of onset of the disorder, duration of illness, and BMI (see Table 2). The BDI scores of subjects with MD were significantly different from those without this diagnosis (37.1 ± 4.5 versus 11.1 ± 4.8; *F* = 550.5; *P* = 0.001), after controlling for age, age of onset of the disorder, duration of illness, and BMI. 

The BDI scores of 408 patients (48.7% of the sample) who were not diagnosed with MD were higher than 10 and so clinically significant; there were statistically significant differences among diagnostic subtypes in this regard (*χ* ^2^ = 9.3859; *P* = 0.02). Considering both the 48.7% of the sample with a BDI score >10 and the 19.5% of MD patients the total percentage of patients with relevant depressive symptomatology is 68.2%.

Patients with MD, irritable mood, anger attacks, or angry outbursts made up 73% of the sample, with no significant differences among diagnostic groups (*χ* ^2^ = 1.321; *P* = 0.724). Moreover, subjects with MD obtained more pathological scores on all STAXI subscales, even after controlling for age, age of onset of the disorder, duration of illness, and BMI, than did patients without MD diagnosis (Table 3). Also subject with clinically significant depressive symptoms (BDI > 10) reported higher STAXI scores than patients without such symptomatology (data not shown).

### 3.3. Eating Psychopathology

After controlling for age, age of onset of the disorder, duration of illness, and BMI, patients with MD showed higher scores on all EDI-2 scales than did those without this diagnosis (Table 4).

### 3.4. Personality

MD patients performed higher scores than those without MD on the HA scale and lower scores on the RD, SD, and C scales of the TCI, even after controlling for age, age of onset of the disorder, duration of illness, and BMI (Table 5).

### 3.5. Logistic Regression

The logistic regression model was significant (*χ* ^2^ = 212.7; df: 36; *P* < 0.001; *R*-square = 0.454). The state anger STAXI subscale (*B* = 0.086; Wald = 13.315; *P* < 0.001), the HA subscale of the TCI (*B* = 0.05; Wald = 5.85; *P* < 0.016), the SD subscale of the TCI (*B* = 0.074; Wald = 8.015; *P* < 0.005), and Ineffectiveness as measured by the EDI-2 (*B* = 0.064; Wald = 5.466; *P* = 0.019) independently correlated with MD. Age, age of onset, ED diagnosis, BMI, episodes of binge-eating and vomiting per week, irritable mood, and other variables measured by the STAXI, TCI, and EDI-2 were not significant.

### 3.6. Correlations and MANOVA

BDI scores correlate significantly directly with HA (*r* = 0.379; *P* < 0.001) and inversely with (*r* = −0,589  *P* < 0.001). Using the MANOVA, HA, and SD differences remain significant even when controlled for BDI scores and for the interaction BDI group (HA: *F* = 75.031; *P* < 0.001; SD: *F* = 227.362; *P* < 0.001). Also the BDI score effect was found significant for both variables (both variables: *P* < 0.001).

## 4. Discussion

### 4.1. Characteristics of Depressive Symptomatology

Data from the present study reported lower MD rates than other studies; such a difference could be due to participants' different stages of illness and it should be also noted that we considered only outpatients while other studies included inpatients.

Significant differences were demonstrated among diagnostic subtypes; patients with purging behaviours (AN-BP and BN) were more likely to be diagnosed with MD when compared to AN and EDNOS. This association is supported by previous researches showing that individuals with purging symptomatology are more likely to show comorbid disorders and greater clinical severity [30, 31]. Also our group in previous studies found a correlation—although not related to diagnosis—with purging symptomatology [5].

Moreover, in our sample MD in ED patients seem typically characterized by irritable mood as measured according to Fava and Kellner criteria [25]. To our knowledge, these results have not been described yet in the literature. We found that depressed ED patients were not inhibited or melancholic, but tended to show angry depression, hostility, aggressiveness, anger attacks, and angry outbursts. In fact, irritability and angry outbursts are approximately twice as prevalent among patients with MD and ED (73%) than among depressed patients without ED, as reported in literature [2, 32]. Results of the STAXI revealed that patients with MD and ED experienced greater difficulty in recognizing, managing, and expressing anger than patients without MD. Also logistic regression considered State Anger as one of the four independent variables correlated to MD diagnosis. Anger problems among those with ED have been well documented in the literature [8, 33, 34], but the role of depressive symptomatology in such difficulties in coping with anger has been rarely considered. Past findings of mood instability deriving from fasting [33], the notorious treatment resistance of ED patients [35], and the presence of self-injurious behaviours [36] highlight other possible sources for angry outbursts and irritability. However, it should be considered the possibility that anger and oppositionalism can originate from depressive symptoms. The importance of evaluating patients with AN and BN for irritable mood is reinforced by the observation that depression and aggressiveness totally mediate the connection between ED and suicidal behavior [37]. Given the correlations between depression and anger, the construct of an anxiety/aggression-driven depression has been proposed to correlate depressive and angry aspects, both related to low serotonergic function [38, 39]. It is noteworthy that MD in ED shows some peculiarities since the course is often protracted, the MD recovery may depend on ED type, and antidepressants are not likely to be as effective as in patients with MD without the ED [40]. Dysphoric traits could underlie such differences in features and course of illness [41].

Considering the BDI, the 48.7% of the sample obtained scores indicating a clinically significant depressive symptomatology (BDI > 10); this datum should be added to the 19.5% of individuals affected by full MD and therefore the total percentage of individuals with relevant depressive symptoms was 68.2%. Moreover, patients with ED were reported to suffer from a wide spectrum of depressive symptoms [42]. Specific characteristics of MD and such a common depressive symptomatology even not meeting MD full criteria highlight the importance of considering also these psychopathological aspects in assessment, monitoring, and treatment of these disorders.

Moreover, also this lager group of depressed patients reported at the STAXI higher scores than ED patients without depressive symptoms. Therefore, previous considerations regarding the group with both ED and DM about high percentage of irritable mood can be extended to depressed patients without an ED.

### 4.2. Depressive Symptomatology and Eating Psychopathology

We found that eating psychopathology, as measured by the EDI-2 scales included in this study, was significantly more severe in patients with comorbid ED and MD than in patients with ED without MD. This correlation between a severe depressive symptomatology and ED severity validated the results of previous studies and confirmed expected hypothesis [2, 3, 30]. Moreover it is well known in literature that eating symptomatology is also associated with depression in women, even among those with no history of threshold-level eating disorder symptomatology [43].

The presence of MD represented an index of clinical severity and/or an indication of the acuity of the ED. Therefore, diagnostic evaluation for MD in patients suffering from AN or BN should be considered, and psychotherapeutic involvement in treatment planning should be included as appropriate, also because these patients are often hopeless about the possibility of change and this should be carefully considered in treatments [37]. Indeed, Ametller et al. [44] have demonstrated that high BDI scores at the first psychiatric assessment represent one of the independent predictors of hospitalization.

The logistic regression analysis showed that the Ineffectiveness subscale of EDI-2 independently predicted MD in the sample. Low self-esteem represents the common core symptom of ED and depression. Thus it could be hypothesized that ED treatments based on cognitive-behavioral therapies focused on low self-esteem [45] can be effective for ED depressed subjects.

Antidepressants might be effective for treating comorbid ED and depression [46]. However, research suggests that psychopharmacological treatment is effective for BN [47], but is of debatable value for AN [40, 48] even to prevent relapse after weight restoration [49].

### 4.3. Depressive Symptomatology and Personality

Patients with both ED and MD were characterized by higher HA and lower scores on the RD, SD, and C scales of the TCI. 

Logistic regression showed that Harm Avoidance and Self-Directedness remained significant after controlling for personal and several clinical variables. These data are consistent with the results of previous studies that have identified these traits as characterizing ED samples when compared to healthy controls [12]. Other studies have shown that these traits persist after recovery from the ED [50] and that they are altered in adolescents at high risk for developing a clinically significant ED [30]. Both in the acute phase and after remission, also patients with MD but without ED obtained high HA and low SD scores on the TCI [17–23]. In fact, such HA and SD alterations are likely to be both state and trait dependent [51]. Also bipolar euthymic patients showed the same pattern [52]. A recent comprehensive review and meta-analysis of the literature investigated the effects of temperament on vulnerability to depression providing evidence that high HA can be associated both with current depressive symptoms and depressive traits [53]. Interestingly, a significant negative change in HA scores has been reported during treatment, and it can be also related to treatment response and recovery. A minority of studies reported also how low Reward Dependence—another temperamental dimension—was associated with depressive symptomatology [53].

This study showed that higher HA and low SD scores were correlated with comorbid MD in ED patients; this correlation was found to be independent of the severity of the ED (as measured by BMI, binge-purging behaviours, and EDI-2 scales), age, age of onset, and duration of illness. Other studies have shown that low SD can predict suicide attempts among ED subjects [35, 54].

ED patients with a personality profile characterized by high HA and low RD, SD, and C represent a subgroup of patients likely to experience feelings of inferiority, inadequacy, unhappiness, anxiety, and dependence [5, 31, 55–57]. It is well known that ED patients with MD represent a substantial group of patients with specific and semi-independent clinical features and that these features require aimed treatments [46, 58]. 

The cross-sectional design of this study makes it difficult to rule out the possibility that high HA scores represented a risk factor or a “scarring effect” for ED and depression on personality [18, 59]. Otherwise it is well known the issue of state dependency of HA and SD from depressive disorder [51]. However, there is growing evidence that high Harm Avoidance levels could represent a trait aspect contributing to vulnerability both to ED [41] and mood disorders [53], and in the present study with the MANOVA analysis we found that the BDI score doesnot completely explain the difference in HA and SD scores between depressed and nondepressed groups. Nevertheless, future research is warranted to perform a longitudinal assessment of the general population to compare premorbid personality traits with those associated with both the ED and depression development during adolescence.

This study is limited by the lack of a control group of healthy subjects or of another clinical population, including patients with other comorbid disorders, and by not considering lifetime comorbidity. On the other hand, one strength of this study is the large sample of patients with MD and ED.

## 5. Conclusions

This study aimed to evaluate comorbidity between ED and MD and the role of personality as predictor of MD in ED. Our data are in line with previous literature since we found a current prevalence of MD of 19.5% with significant differences among diagnostic subtypes since patients with purging behaviours were more likely to be affected by MD. Irritability was found to be a feature of MD in ED with rates of irritability and angry outbursts twice as prevalent among patients with MD and ED (73%) than among depressed patients without ED as reported in the literature. Considering the BDI, the 48.7% of the sample obtained scores indicating a clinically significant depressive symptomatology (BDI > 10). The eating psychopathology, as measured by the EDI-2 scales, was significantly more severe in patients with MD comorbidity. With regard to personality dimensions, patients with ED and MD showed higher Harm Avoidance and lower scores on the Reward Dependence, Self-Directedness, and Cooperativeness scales of the TCI. The personality dimensions of high HA and low SD could be risk factors in the development of Major Depression in ED individuals because the differences between depressed and non-depressed groups remain significant even after controlling for the BDI score and BDI group interaction.

Clinicians should carefully evaluate in patients with Eating Disorders their depressive symptomatology and the role of anger and personality to provide effective treatments tailored to person and not based only on symptomatology [60].

## Figures and Tables

**Table 1:** Sociodemographic characteristics of the sample.

	Total sample (*n* = 838)
Female	100%
Caucasian	100%

**Table 2:** Clinical features of the sample.

	ED without MD	ED with MD	*t*	*P*
Age	28.54 ± 9.36	29.68 ± 10.08	−1.376	0.169
Age of onset	19.93 ± 7.85	20.24 ± 8.18	−0.450	0.653
Duration of illness (months)	103.49 ± 94.90	113.66 ± 103.03	−1.207	0.228
BMI: total group	18.88 ± 3.72	19.33 ± 3.72	−1.394	0.164

ED: Eating Disorder; MD: Major Depression; BMI: body mass index.

**Table 3:** State-Trait Anger Expression Inventory (STAXI).

	ED without MD	ED with MD	*t*	*P*
S-Anger	13.85 ± 5.53	20.50 ± 8.99	−11.394	0.001
T-Anger	22.14 ± 6.56	25.91 ± 6.46	−6.469	0.001
T-Anger/T	8.00 ± 3.17	9.57 ± 3.41	−5.323	0.001
T-Anger/R	10.37 ± 4.04	11.85 ± 2.88	−4.191	0.001
AX-In	18.90 ± 5.70	22.21 ± 4.98	−6.508	0.001
AX-Out	16.02 ± 5.08	17.66 ± 5.48	−3.494	0.001
AX-Con	20.51 ± 6.01	18.10 ± 6.40	4.330	0.001
AX-Ex	30.40 ± 11.33	37.57 ± 10.57	−7.020	0.001

ED: Eating Disorder; MD: Major Depression; S-Anger: State-anger; T-Anger: Trait-anger; AX-In: Anger Expression-In; AX-Out: Anger Expression-Out; AX-Con: Anger Expression Control; AX-Ex: Anger Expression.

**Table 4:** Eating Disorder Inventory-2 (EDI-2).

	ED without MD	ED with MD	*t*	*P*
DT	11.24 ± 7.27	15.83 ± 6.29	−7.400	0.001
B	5.91 ± 5.56	8.49 ± 6.38	−5.138	0.001
BD	12.33 ± 7.72	18.04 ± 6.71	−8.657	0.001
I	8.33 ± 6.37	17.46 ± 6.88	−16.125	0.001
P	5.25 ± 4.13	7.02 ± 4.45	−4.834	0.001
ID	5.57 ± 4.46	9.30 ± 4.87	−9.359	0.001
IA	9.16 ± 6.60	15.54 ± 7.33	−10.802	0.001
MF	6.42 ± 5.03	8.95 ± 6.03	−5.514	0.001
A	6.69 ± 4.25	9.91 ± 4.60	−8.517	0.001
IR	6.43 ± 5.75	12.59 ± 6.76	−11.822	0.001
SI	7.10 ± 5.03	12.00 ± 4.38	−11.399	0.001

ED: Eating Disorder; MD: Major Depression; DT: drive for thinness; B: bulimia; BD: body dissatisfaction; I: Ineffectiveness; P: perfectionism; ID: interpersonal distrust; IA: interoceptive awareness; MF: maturity fears; A: Asceticism; IR: impulse regulation; SI: social insecurity.

**Table 5:** Temperament and Character Inventory (TCI).

	ED without MD	ED with MD	*t*	*P*
NS	20.70 ± 9.75	19.53 ± 6.34	1.450	0.148
HA	22.00 ± 9.35	26.88 ± 5.69	−6.329	**0.001**
RD	15.53 ± 5.50	14.31 ± 3.65	2.675	**0.008**
P	5.28 ± 5.14	4.66 ± 2.03	1.498	0.134
SD	23.08 ± 8.30	15.38 ± 6.29	10.983	**0.001**
C	30.75 ± 7.34	27.10 ± 7.50	5.630	**0.001**
ST	13.86 ± 7.45	13.85 ± 6.72	0.002	0.998

ED: Eating Disorder; MD: Major Depression; NS: novelty seeking; HA: harm avoidance; RD: reward dependence; P: persistence; SD: Self-Directedness; C: cooperativeness; ST: Self-transcendence.
